# Acylated Aminooligosaccharides with Inhibitory Effects against *α*-Amylase from *Streptomyces* sp. HO1518

**DOI:** 10.3390/md16110403

**Published:** 2018-10-23

**Authors:** Hai-Li Liu, Heng-Chao E, Ding-An Xie, Wen-Bo Cheng, Wan-Qi Tao, Yong Wang

**Affiliations:** 1 Key Laboratory of Synthetic Biology, CAS Center for Excellence in Molecular Plant Sciences, Institute of Plant Physiology and Ecology, Shanghai Institutes for Biological Sciences, Chinese Academy of Sciences, Shanghai 200032, China; hlliu@sibs.ac.cn (H.-L.L.); ehengchao@126.com (H.-C.E.); chengwenbo@sippe.ac.cn (W.-B.C.); 2 University of Chinese Academy of Sciences, Beijing 100039, China; 3 College of Food Science and Engineering, Ocean University of China, Shanghai 201306, China; dinganxie@163.com; 4 School of Life Sciences, University of Liverpool, Liverpool L69 3BX, UK; W.Tao3@student.liverpool.ac.uk

**Keywords:** *Streptomyces* sp. HO1518, acylated aminooligosaccharides, porcine pancreatic *α*-amylase (PPA) inhibitor

## Abstract

Five new acylated aminooligosaccharides (**1**–**5**), together with one known related analogue (**6**), were isolated from *Streptomyces* sp. HO1518. Their structure was identified by extensive spectroscopic analysis, including 1D and 2D NMR data and high resolution electrospray ionization mass spectrometry (HRESIMS), and by comparison with those reported in the literature. All of the new compounds showed more promising porcine pancreatic *α*-amylase (PPA) inhibitory activities than the clinical drug acarbose, indicating them as potential pharmaceutical drug leads toward type II diabetes.

## 1. Introduction

*α*-Amylase, belonging to glycoside hydrolase family 13 (GH 13), is widely found in organisms, including plants, animals, bacteria, and fungi [1]. It is an endoamylas, which catalyzes the hydrolysis of *α*-(1 → 4)-d-glucosidic bind of starch, amylase, amylopectin, glycogen, and various maltodextrins into smaller oligomers [2]. The inhibitors of *α*-amylase are of pharmacological importance, as *α*-amylase is considered as an attractive target for treating elevated post-prandial blood glucose levels resulting in type II insulin-independent diabetes, obesity, and other related secondary symptoms [3]. The well-known acarbose, originated from *Actinoplanes* species, is recognized as one of the most clinically important *α*-amylase inhibitor [4]. It is a member of the aminooligosaccharides family exclusively produced by microorganisms, particularly soil bacteria of the order of Actinomycetes. Structurally, aminooligosaccharides contain a single or repeated pseudo-trisaccharide core(s) with D-glucose units attached to the reducing and non-reducing end through *α*-(1 → 4) glycosidic bond [4,5]. Pseudo-trisaccharide core is formed by an acarviosine moiety and a D-glucopyranose group through an *α*-(1 → 4) quinovosidic bond; acarviosine is composed of an unsaturated C_7_N aminohexitol (valienamine) unit and a 4,6-dideoxy-d-glucopyranose (4-deoxy-d-quinovopyranose) unit; and the amino-hexitol bond is defined as pseudo-glycosidic bond. Therefore, aminooligosaccharides are termed as acarviostatins followed by a Roman numeral and two numbers, i.e., acarviostatin I01 (acarbose). Acarvios originates from the acarviosine core; the Roman numeral represents the number of the pseudo-trisaccharide cores; the middle digit indicates the number of glucose residues at the no-reducing end and the last digit infers the number of glucose residues at the reducing end. Generally, an extensive MS study is helpful to discriminate the structure of aminooligosaccharides, due to their characteristic positive-ion ESIMS/MS fragmentation pattern [6,7,8,9,10,11,12,13,14,15,16]. The formation of bi and yj fragment ions is typical of glycosidic bond dissociation of protonated oligosaccharides. Every glycosidic bond could be dissociated, including the pseudo-glycosidic bond within the acarviosin moiety. Moreover, the cleavage of the quinovosidic bond in the pseudo-trisaccharide core happened more readily than that of an ordinary glycosidic bond in the molecule. Until now, guided by the MS study, hundreds of aminooligosaccharides have been quickly identified, including trestatins [6,7], isovalertatins [8,9], butytatins [10], acarviostatins [11,12,13,14,15], SF638-1 [16], as well as artificial acarbose analogues (i.e., G_6_-Aca, G_12_-Aca) [2]. Many have shown more potent porcine pancreatic *α*-amylase (PPA) activities than acarbose, among which acarviostatins III03 [11] was the most effective PPA inhibitor known to data. It was reported that the acarviosine unit was postulated to be essential for its biological activity [17]. In addition, the number of the pseudo-trisaccharide cores along with the D-glucose residues also affected the PPA inhibitory activity [18].

**Figure 1:**
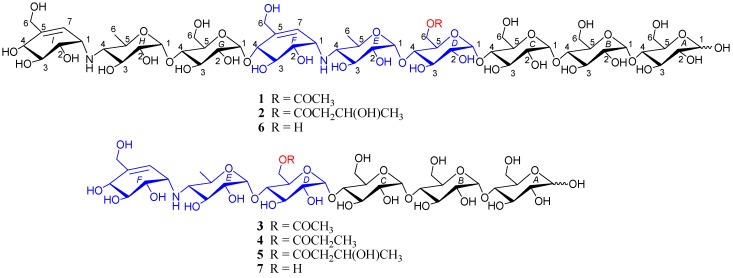
Structure of compounds **1**–**7**.

As part of our ongoing search for novel biologically active natural products from marine actinomycetes [19,20,21,22], we chemically investigated *Streptomyces* sp. HO1518 from sediments collected off Yellow Sea, close to Rizhao, Shandong province, China. Fractionation of the resins-absorbed extract of the liquid fermentation broth of the strain led to the isolation of five new metabolites **1**–**5** (Figure 1), namely acylated aminooligosaccharides, D6-*O*-acetyl-acarviostatin II03 (**1**), D6-*O*-*β*-hydroxybutyryl-acarviostatin II03 (**2**), D6-*O*-acetyl-acarviostatin I03 (**3**), D6-*O*-propionyl-acarviostatin I03 (**4**), and D6-*O*-*β*-hydroxybutyryl-acarviostatin I03 (**5**), as well as one known related analogue, acarviostatin II03 (**6**). This paper describes the isolation, structural elucidation, and PPA inhibitory activity of these metabolites.

## 2. Results and Discussion

A large-scale fermentation broth of *Streptomyces* sp. HO1518 was collected and absorbed with Amberlite XAD-16 resins to produce a crude extract. The subsequent fractionation by repeated column chromatography over ODS-C_18_ and semi-preparative reversed-phase HPLC afforded the pure acylated aminooligosaccharides **1**–**5**, together with one known related one (**6**). The known compound was identified as acarviostatin II03 by comparison of its [*α*]_D_ value {**6**: [α]${}_{D}^{22}$ + 148.7 (*c* 0.59, H_2_O) [α]${}_{D}^{18}$ + 165.0 (*c* 0.1, H_2_O)] [11]}, positive-ion ESIMS/MS fragmentation pattern and 1D NMR data with those reported in the literature.

**Figure 2:**
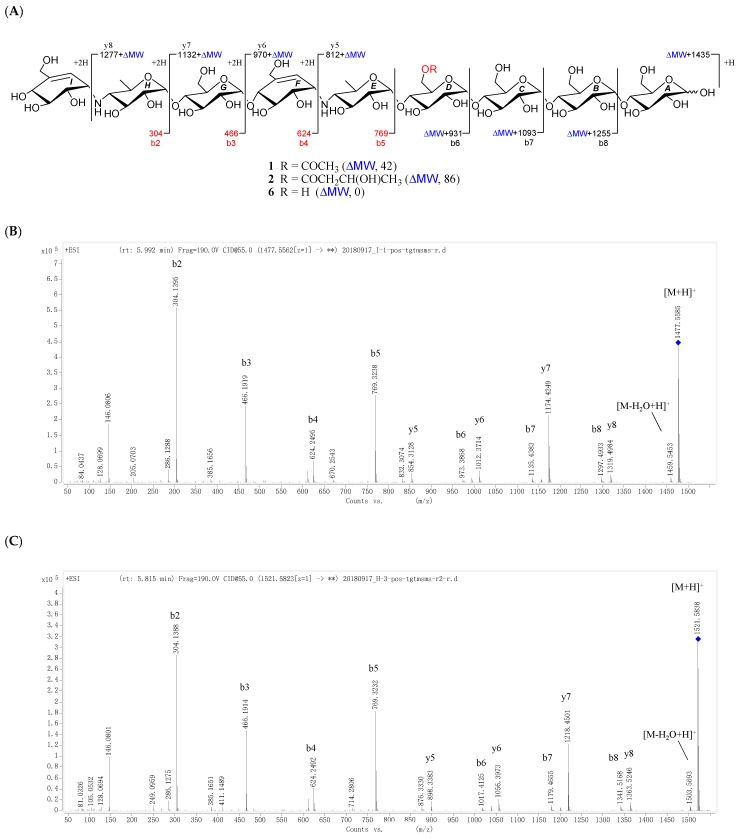

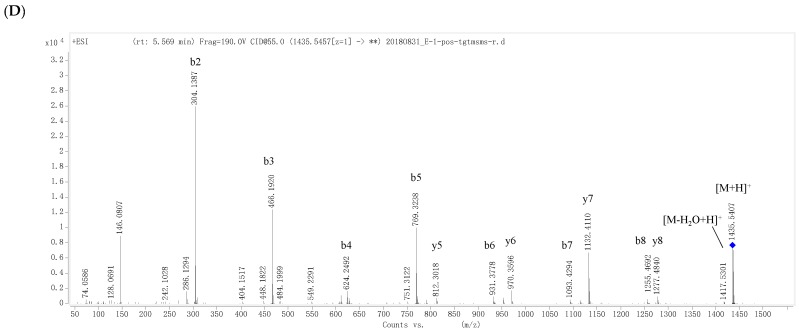
Positive-ion HRESIMS/MS fragmentation pattern and spectra of **1**, **2** and **6**. (**A**) Positive-ion HRESIMS/MS fragmentation pettern of **1**, **2** and **6**; (**B**–**D**) Positive-ion HRESIMS/MS spectra of **1**, **2** and **6**.

**Figure 3:**
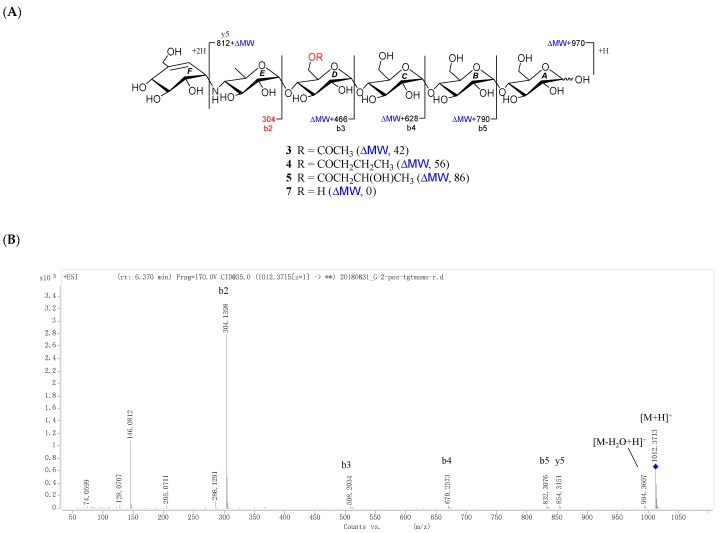

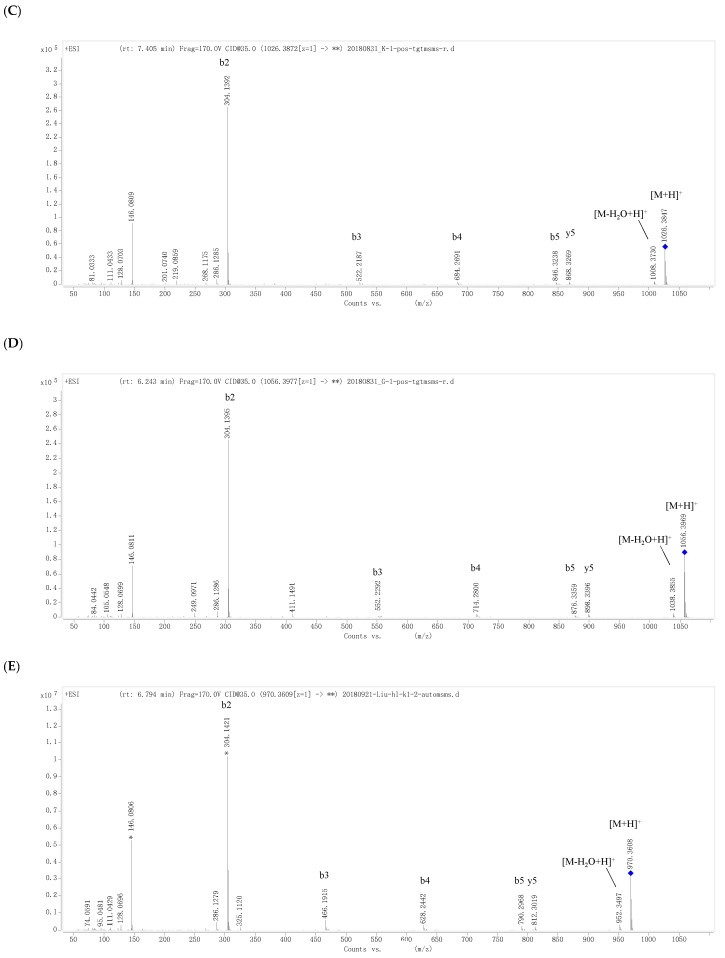
Positive-ion HRESIMS/MS fragmentation pattern and spectra of **3**–**5** and **7**. (**A**) Positive-ion HRESIMS/MS fragmentation pattern of **3**–**5** and **7**; (**B**–**E**) Positive-ion HRESIMS/MS spectra of **3**–**5** and **7**.

All the new compounds showed similar IR absorptions indicative of the presence of oligosaccharide (~3300 and ~1000 cm^−1^), ester carbonyl (~1720 cm^−1^), and double bond(s) (~1640 cm^−1^) and they also exhibited similar positive-ion HRESIMS/MS fragmentation pattern, which further divided them into two groups. As for **1** and **2** (Figure 2, Figures S19 and S41), their common fragment ion peaks at *m*/*z* 304 (b2), 466 (b3), 624 (b4), and 769 (b5) were immediately discriminated, the same as those of co-occurring acarviostatin II03 (**6**), while the fragment ion peaks at *m*/*z* y5–y8 and b6–b8 in **1** and **2**, along with their pseudo-molecular ion peaks, were 42 and 86 mass units more than those of **6** (Figure 2 and Figure S105), respectively. According to **3**–**5** (Figure 3, Figures S60, S79 and S98), they possessed the sole mutual fragment ion at *m*/*z* 304 (b2), with their primary MS ion peaks and all the other secondary MS/MS ion peaks at *m*/*z* y4, y5 and b3–b5 in **3**–**5** being 42, 56, and 86 mass units more than those of the model acarviostatin I03 (**7**) (Figure 3 and Figure S107), previously obtained from the soil *Streptomyces coelicoflavus* ZG0656 [11], respectively. Moreover, their NMR data was also reminiscent of those of acarviostatin II03 (**6**) and acarviostatin I03 (**7**). In fact, **1** and **2** like co-occurring **6** exhibited the same acarviostatin II03-type core, with their acyl units linked to the C-**D**6 ester differing, while **3**–**5** showed the same acarviostatin I03-type core as in model **7**, differ from each other also in acyl units linked to the C-**D**6 ester. Further alkaline hydrolysis of **1** and **2** afforded precursor **6**, while treatment of **3**–**5** with methanolic ammonium hydroxide provided precursor **7**.

**Figure 4:**
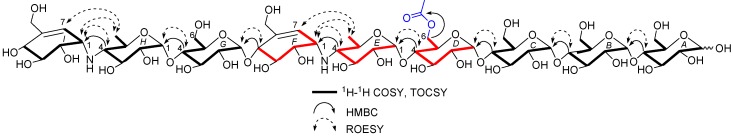
Key ^1^H-^1^H COSY, TOCSY, HMBC, and ROESY correlations for **1**.

D6-*O*-acetyl-acarviostatin II03 (**1**) was isolated as a white amorphous powder, and its molecular formula, C_58_H_9__6_N_2_O_41_ was established by its HRESIMS of pseudo-molecular ion peak [M + H]^+^ (*m*/*z* 1477.5564, cald 1477.5561), implying 12 site of unsaturation. The ^13^C NMR (Table 1) and DEPT spectra revealed the following carbon types: Three methyl, seven *sp*^3^ methylene, two *sp*^2^ methine, forty-three *sp*^3^ methine, and three *sp*^2^ quaternary carbons. Three of the twelve degrees of unsaturation were accounted for by the two tri-substituted double bonds (*δ* 139.4 and 129.2; *δ* 141.8 and 126.6) and one carbonyl group (*δ*_C_ 177.0) observed in the ^13^C NMR spectrum. Consequently, **1** should have nine rings (residues **A**–**I**). The NMR data of **1** was similar to those of co-occurring known acarviostatin II03 (**6**), showing the characteristic terminal unit of acarviosin in the non-reducing end, the typical reducing D-glucose terminus, as well as an inner acarviosin moiety. In fact, careful comparison of the NMR data of **1** and **6** revealed that the only difference between them resided in an additional acetyl group resonating at *δ*_H_ 2.17 (3H, s) and *δ*_C_ 177.0 (qC) and 23.1 (CH_3_) in **1**, in agreement with the 42 mass units difference between them, while the rest of the structure of **1** was the same as in **6**. Due to the effect of the acetyl group, a series of ^1^H NMR data changes associated with the inner pseudo-trisaccharide core (residues **D**–**F**) were clearly detected in **1**. The usually overlapped methylene signal in the D-glycopyranose residue **D** was obviously downfield shift from *δ* 3.84 (2H, m, H_2_-**D**6) in **6** to *δ* 4.43 (1H, brd, *J* = 12.5 Hz, H-**D**6a) and 4.24 (1H, m, H-**D**6b) in **1**, whereas the anomeric proton signal (H-**E**1) in the residue **E** within the inner acarviosine moiety was slightly moved to highfield from *δ* 5.33 (1H, d, *J* = 3.5 Hz) in **6** to *δ* 5.30 (1H, d, *J* = 3.5 Hz) in **1**. However, the characteristic olefinic proton signal at *δ* 5.98 (1H, d, *J* = 4.5 Hz, H-**F**7) in the residue **F** within the inner acarviosine moiety was unaffected. When selective irradiated the three independent ^1^H signals resonating at *δ* 4.43 (H-**D**6a) in residue **D**, *δ* 5.33 (H-**E**1) in residue **E**, and *δ* 5.98 (H-**F**7) in residue **F**, respectively, the three self-spin systems of the inner pseudo-trisaccharide core were quickly acquired by the 1D-selective TOCSY experiments (Figure 4) with a 120 ms spin lock time. Subsequently, their corresponding ^13^C NMR data were accurately obtained by the following HSQC-TOCSY (60 ms spin lock time) combined with HSQC experiments. In particular, the significant downfield shift of C-**D**6 (*δ* 66.5 in **1** vs. *δ* 63.5 in **6**), C-**D**4 (*δ* 80.6 in **1** vs. *δ* 79.8 in **6**), and C-**E**1 (*δ* 103.4 in **1** vs. *δ* 102.7 in **6**), accompanied with the obvious highfield shift of C-**D**5 (*δ* 74.0 in **6** to *δ* 71.7 in **1**), with respect to those of **6**, were in accordance with the observations appeared in its ^1^H NMR spectrum. From these evidences, the location of acetyl ester linkage was unambiguously determined to be at C-**D**6, which was further confirmed by the distinct HMBC correlations (Figure 4) from H_2_-**D**6 to the acetyl carbonyl and from its TOCSY coupled H-**D**4 (*δ*_H_ 3.66, m, 1H) to the C-**E**1 (*δ* 103.4). Moreover, the significant HMBC correlations from H-**D**4 to C-**E**1 and from H-**E**4 (*δ*_H_ 2.46, t, *J* = 9.0 Hz, 1H) to C-**F**1 (*δ*_C_ 57.8) and the obvious ROESY correlations (Figure 4) of H-**D**4/H-**E**1, H-**E**4/H-**F**1, Me-**E**6/H-**F1**, and Me-**E**6/H-**F7** jointed the residues **E** with **D** and **F** in the inner pseudo-trisaccharide core. This is consistent with the positive-ion HRESIMS/MS data (Figure 2A,B and Figure S19), showing a series of fragment ions at *m*/*z* 1319 (y8), 1174 (y7), 1012 (y6), 854 (y5), 769 (b5), 466 (b3), and 304 (b2) formed by the cleavage of quinovosidic, pseudoglycosidic, and glycosidic bonds. The complete ^1^H and ^13^C assignment of the **1** was inferred by combing information from the 1D-selective TOCSY, HSQC, HSQC-TOCSY, and HMBC experiments. Finally, the configuration of the glycosidic bonds of **1** was deduced to be *α*-(1 → 4), the same as that of **6**, confirmed by the ^1^H-^1^H coupling constants of the anomeric protons and the NOESY experiment and proved by the chemical conversion between **1** and **6**. Therefore, the structure of D6-*O*-acetyl-acarviostatin II03 (**1**) was established, as shown in Figure 1.

**Figure 5:**
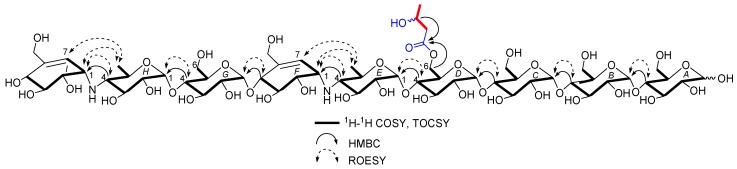
Key ^1^H-^1^H COSY, TOCSY, HMBC, and ROESY correlations for **2**.

The HRESIMS of D6-*O*-*β*-hydroxybutyryl-acarviostatin II03 (**2**) established the molecular formula, C_60_H_100_N_2_O_42_ [*m*/*z* 1521.5829 [M + H]^+^ (calcd. for C_60_H_101_N_2_O_4__2_, 1521.5823)], indicating 44 mass units more than that of **1**. Comparison of the overall ^1^H and ^13^C NMR data of **2** with those of **1** revealed that the difference can be recognized in the absence in **2** of the acetyl group replaced by a *β*-hydroxybutyryl group [*δ*_C_ 176.6; *δ*_H_ 2.67 (dd, *J* = 15.0, 4.5 Hz), 2.59 (dd, *J* = 15.0, 8.0 Hz)/*δ*_C_ 45.9; *δ*_H_ 4.27 (m)/*δ*_C_ 67.3; and *δ*_H_ 1.26 (d, 6.4 Hz)/*δ*_C_ 24.8 in **2**]. The presence of the *β*-hydroxybutyryl group was supported by the newly appeared CH_3_CH(OH)CH_2−_ spin-spin coupling system in the TOCSY spectrum (Figure 5) and by the HMBC correlation (Figure 5) between the oxy-methine (*δ*_H_ 4.27) and the ester carbonyl (*δ*_C_ 176.6, qC). Accordingly, the ^13^C NMR data in the inner pseudo-trisaccharide core (residues **D**–**F**) in **2** remained unaffected, although different acyl groups, *β*-hydroxybutyryl group in **2** vs. acetyl group in **1**, were esterified at C-**D**6. Moreover, the similar ^13^C NMR data changes in **2** around the residue **D** (C-**D**4, +1.0 ppm; C-**D**5, −2.2 ppm; C-**D**6, +3.0 ppm in **2**) and its adjacent residue **E** (C-**E**1, +0.7 ppm in **2**), with respect to their common precursor **6**, the distinct HMBC correlation between H_2_-**D**6 [*δ*_H_ 4.50 (dd, *J* = 12.5, 2.0 Hz) and 4.26 (m)] and the ester carbonyl, and a series of positive-ion HRESIMS/MS peaks (Figure 2A,C and Figure S41) at *m*/*z* 1363 (y8), 1218 (y7), 1056 (y6), 898 (y5), 769 (b5), 466 (b3), and 304 (b2) further supported the assignment. Analogously to **1** and **6**, the configuration of the glycosidic bonds was determined to be *α*-(1 → 4) by analysis of the ^1^H-^1^H coupling constants of the anomeric protons and by interpretation of its NOESY experiment (Figure 5), which was confirmed by the alkaline hydrolysis of **2** to **6**. Thus, the structure of D6-*O*-*β*-hydroxybutyryl-acarviostatin II03 (**2**) was determined as depicted.

D6-*O*-acetyl-acarviostatin I03 (**3**) was obtained as an optical active white amorphous powder. Its molecular formula, C_39_H_65_NO_29_, was suggested by a sodiated molecular ion peak at *m*/*z* 1012.3713 [M + H]^+^ (calcd for C_39_H_66_NO_29_ 1012.3715). The ^13^C NMR (Table 2) spectrum revealed 39 carbon resonances, which were classified by DEPT and HSQC experiments as a ester carbonyl (*δ* 177.0), a tri-substituted double bond (*δ* 142.3 and 126.1), two methyls, five *sp*^3^ methylenes, and twenty-nine *sp*^3^ methines. The olefin and carbonyl groups accounted for two of the eight degrees of unsaturation, therefore, **3** should be in hexacyclic ring system (residues **A**–**F**). Its ^1^H and ^13^C NMR data (Table 2) closely resembled those of **1**, suggesting that **3** possessed a single pseudo-trisaccharide core with three D-glucose units on the reducing end and also an acetyl ester at C-**D**6, in analogy to **1**. In fact, **3** differs from **1** only by the absence of the repeated pseudo-trisaccharide core in the non-reducing end in **1**, in agreement with 465 mass units less than that of **1**, which was supported by the disappearance of the characteristic NMR signals of residues **F**–**H** in **1** resonating at *δ*_H_ 3.53 (H-**F**1)/*δ*_C_ 57.8 (C-**F**1), *δ*_H_ 4.24 (H-**F**4)/*δ*_C_ 79.1 (C-**F**4), *δ*_H_ 4.22, 4.14 (H_2_-**F**6)/*δ*_C_ 64.9 (C-**F**6), *δ*_H_ 5.98 (H-**F**7)/*δ*_C_ 129.2 (C-**F**7), and *δ*_C_ 139.4 (C-**F**5); *δ*_H_ 5.38 (H-**G**1)/*δ*_C_ 100.4 (C-**G**1) and *δ*_H_ 3.86 (H_2_-**G**6)/*δ*_C_ 63.5 (C-**G**6); and *δ*_H_ 5.33 (H-**H**1)/*δ*_C_ 103.4 (C-**H**1), *δ*_H_ 2.48 (H-**H**4)/*δ*_C_ 67.8 (C-**H**4), and *δ*_H_ 1.35 (Me-**H**6)/*δ*_C_ 20.2 (C-**H**6), respectively. Moreover, the ^1^H and ^13^C NMR spectra of **3** were almost superimposable on those of the model known acarviostatin I03 (**7**) [11], with the exception of an additional acetyl group [*δ*_C_ 177.0 (qC); *δ*_H_ 2.16 (3H, s)/*δ*_C_ 23.1 (CH_3_)] in **3**. The distinct HMBC correlation from H_2_-**D**6 [*δ*_H_ 4.43 (1H, dd, *J* = 12.5, 2.0 Hz) and 4.24 (1H, brd, *J* = 12.5 Hz)] to the acetyl carbonyl and the characteristic HRESIMS/MS fragment ions (Figure 3A,B and Figure S60) at *m*/*z* 854 (y5), 832 (b5), 670 (b4), 508 (b3), and 304 (b2) allowed to locate the acetyl group at OH-**D**6, thus completely defining the structure of **3**. Finally, the small ^1^H-^1^H coupling constants of the anomeric protons and its ROESY spectrum indicated the glycosidic bonds to be *α*-(1 → 4), identical to those in **7**, which was supported by chemical conversion of **3** to **7**. Therefore, compound **3** is an acetyl derivative of **7**, namely as D6-*O*-acetyl-acarviostatin I03.

D6-*O*-propionyacarviostatin I03 (**4**) had a molecular formula, C_40_H_67_NO_29_, as determined by HRESIMS [*m*/*z* 1026.3872 [M + H]^+^, corresponding to C_40_H_68_NO_29_ (calcd 1026.3872)], 14 mass units more than that of **3**. Its ^1^H and ^13^C NMR data (Table 2) are similar to those of **3**, except for the replacement of the acetyl group in **3** by a propionyl group [*δ*_C_ 180.3 (qC), *δ*_H_ 2.48 (2H, q, *J* = 7.5 Hz)/*δ*_C_ 30.0 (CH_2_), *δ*_H_ 1.13 (3H, t, *J* = 7.5 Hz)/*δ*_C_ 11.1 (CH_3_)] in **4**. The presence of propionyl group was corroborated by the ^1^H-^1^H COSY correlation between *δ*_H_ 2.48 and *δ*_H_ 1.13. The significant HMBC correlation from H_2_-**D**6 [(*δ*_H_ 4.44 (1H, dd, *J* = 12.5, 2.0 Hz) and 4.24 (1H, brd, *J* = 12.5 Hz)] to the propionyl carbonyl placed it at C-**D**6, which was supported by a series of typical fragment ions at *m*/*z* 868 (y5), 846 (b5), 684 (b4), 522 (b3), and 304 (b2) in its positive-ion HRESIMS/MS (Figure 3A,C and Figure S79). The configuration of glycosidic bonds in **4** were deduced to be *α*-(1 → 4) by analysis of the coupling constants of the anomeric protons and its ROESY spectrum, the same as those in **3,** thereby completing the structure of **4**.

D6-*O*-*β*-hydroxybutyryl-acarviostatin I03 (**5**) had the molecular formula of C_41_H_69_NO_30_ based on HRESIMS pseudo-molecular ion peak at *m*/*z* 1056.3976 [M + H]^+^ (cald for C_41_H_70_NO_30_, 1056.3977). A comparison of the overall ^1^H and ^13^C NMR data (Table 2) of **5** with those of **3** and **4** suggested that the three compounds had the same acarviostatin I03-type basic core. In fact, the only difference among them existed at C-**D**6, where the acetyl group in **3** or the propionyl group in **4** was replaced by the *β*-hydroxybutyryl group in **5**. Moreover, the *β*-hydroxybutyryl group at C-**D**6 was further confirmed by comparison of its NMR data with those of **2**, which showed great similarity due to the same partial structure between **2** and **5** except for the absence of the repeated pseudo-trisaccharide unit in **2**. All the 1D-selective TOCSY, HSQC, HSQC-TOCSY, and HMBC experiments together with the HRESIMS/MS fragmentation pattern (Figure 3A,D and Figure S98) unambiguously determined the structure of **5**. Analogously to **2**–**4**, the configuration of the glycoside bonds in **5** was deduced to be *α*-(1→4) by analysis of the ^1^H-^1^H coupling constants of the anomeric protons and by interpretation of its ROESY experiment. On the basis of above evidences, the structure of *β*-hydroxybutyryacarviostatin I03 (**5**) was determined as depicted.

Additionally, both **2** and **5** possess a common *β*-hydroxybutyryl side chain. There is a chiral carbon at the *β* position of the carbonyl function. To determine its absolute chemistry, the alkaline hydrolysis method followed by methyl esterification reaction [23,24] was applied to **2** and **5**. Unfortunately, the efforts for obtaining its corresponding methyl 3-hydroxybutyrate failed, mainly due to the limit amount of **2** and **5** obtained. Therefore, the absolute configuration in the side chain still remains undetermined.

Aminooligosaccharides are of particular interest, due to their unique structures and promising *α*-amylase inhibitory activities. Although aminooligosaccharides continues to be found in recent years [14,15], acylated acarviostatins are relatively rare. Actually, to our knowledge, only eleven isovalertatins [8,9] and two butytatins M03 and M13 [10] from soil *Streptomyces luteogrise*, have been isolated and purified to data. All actylated acarviostatins described in the current work are C-**D**6 ester. The discovery of new acylated acarviostatins **1**–**5** from marine *Streptomyces* strains has added to an extremely diverse and complex array of aminooligosaccharides, which is still rapidly expanding.

As mentioned above that the inhibitors of *α*-amylase are of pharmacological importance against diabetes, obesity, and hyperlipidema. In our screening program to search for *α*-amylase inhibitors from the Yellow Sea marine actinomycetes, we evaluated compounds **1**–**5** for their inhibitory activity against PPA and the results revealed that all new isolates showed promising PPA inhibitory activities with IC_50_ values ranging from 0.03 to 0.70 µM (Table 3), of which **1** (0.029 μM) and **2** (0.049 μM), being similar to **6**, were ca. 540- and 320-fold, while **3**–**5** were ca. 23- to 80-fold, stronger than the positive control acarbose (15.7 μM). Interestingly, the *β*-hydroxybutyryl group substituted in ring **D** slightly increased the PPA inhibitory activity in **5** compared to **3** and **4**, but it simultaneously somewhat reduced the PPA inhibitory activity in **2** compared to **1** and **6**. This phenomenon suggested that the number of the pseudo-trisaccharide cores and glucose residues, as well as the bulk of the acyl groups maybe mutually affect the PPA activities. To the best of our knowledge, this is the first report of the inhibitory activities of acylated acarviostatins against PPA.

## 3. Materials and Methods

### 3.1. General Experimental Procedures

Optical rotations were measured on a Autopol VI automatic polarimeter (Rudolph Research Analytical, Hackettstown, NJ, USA). UV spectra were recorded on a JASCO V-550 UV/VIS spectrophotometer (JASCO, Tokyo, Japan). IR spectra were recorded on a JASCO FT/IR-480 plus Fourier transform infrared spectrometer (JASCO, Tokyo, Japan). ^1^H and ^13^C NMR spectra were recorded at 298 K on a Bruker AVANCE-400 spectrometer ((Bruker BioSpin, Rheinstetten, Germany, 400 MHz for ^1^H and 100 MHz for ^13^C) and a Bruker AVANCE-500 spectrometer (Bruker BioSpin, Rheinstetten, Germany, 500 MHz for ^1^H and 125 MHz for ^13^C). The concentrations for NMR experiments were 18.0 mg/mL for 1, 2.8 mg/mL for 2, 6.0 mg/mL for 3, 20.0 mg/mL for 4, and 4.0 mg/mL for 5. The total acquired times for 1–5 were 48.7, 66.1, 51.3, 66.9, and 56.3 h, respectively. The chemical shifts were given in *δ*, with the TMSP-2,2,3,3-*d*_4_ signals in D_2_O solvent as an internal standard, and the coupling constants (*J*) were in Hz. Spectra assignment was aided by the recording of DEPT-135, HSQC, HMBC, ^1^H-^1^H COSY, 1D selected TOCSY (120 ms mixing time), HSQC-TOCSY (60 ms mixing-time), 2D TOCSY (80 ms mixing time), and ROESY (500 ms mixing time). HRESIMS data were obtained on a Thermo Q Exactive mass spectrometer (Thermo Fisher Scientific, Waltham, MA, USA). ESIMS/MS data were acquired on an Agilent Q-TOF 6545 mass spectrometer (Agilent Technologies, Santa Clara, CA, USA). Analytical HPLC was performed on a Thermo ultimate 3000 (Thermo Fisher Scientific, Waltham, MA, USA) with Alltech 3300 ELSD detector by using an ODS column (Phenomenex/Luna C18(2) 100A, *φ* 5 μm, 4.6 × 250 mm). Semi-preparative HPLC was performed on a Thermo ultimate 3000 (Thermo Fisher Scientific, Waltham, MA, USA) with VWD detector by using an ODS column (TSK-gel 100 V C_18_, *φ* 5 μm, 10 × 250 mm) at 210 nm. Column chromatography (CC) was carried out on ODS-C_18_ (60–80 mesh; YMC Ltd., Kyoto, Japan). TLC was carried out on Silica gel GF254 (YanTai Chemical Inst., Yantai, China) plates. All solvents used in CC were of analytical grade (Sinopharm Chemical Reagent Co., Ltd., Beijing, China).

### 3.2. Strain Isolation and Cultivation

The actinomycetes *Streptomyces* sp. HO1518 was isolated from the sediments collected off Yellow Sea, close to the City of Rizhao, Shandong Province, China, at a depth of 50–100 m, in summer 2010 [19]. The isolate was identified as *Streptomyces* sp. by its morphological characteristics and the voucher specimen (No. M2018176) was deposited in the China Center for Type Culture Collection (CCTCC), Wuhan University. The strain was cultivated on MS agar plate consisting of 20 g/L soybean flour, 20 g/L mannitol, 20 g/L agar powder, and deionized water at 28 °C for 7 days. Then small agar plugs with mycelia were inoculated into five 1 L-Erlenmeyer flasks, each containing 400 mL TSB liquid medium, which were cultivated at 28 °C with 200 rpm/min. After 3 days of fermentation, 40 mL of the seed cultures were inoculated into 50 flasks with 400 mL phamamedia liquid medium consisting of 10 g/L phamamedia powder, 10 g/L soluble starch, 12 g/L glucose, 5 g/L corn extract powder, and artificial seawater (ASW [per liter]: 24.5 g NaCl, 4 g Na_2_SO_4_, 0.7 g KCl, 0.1 g KBr, 0.04 g SrCl_2_·6H_2_O, 5 g MgCl_2_·6H_2_O, 1 g CaCl_2_, 0.2 g NaHCO_3_, 0.03 g H_3_BO_3_, and 0.004 g NaF), pH 7.2, and fermented on a rotary shaker with 200 rpm/min at 28 °C for 7 days.

### 3.3. Extraction and Isolation

The entire filtrate of the fermented broth (20 L) was harvested, added with Amberlite XAD-16 resins and continue shaked at 200 rpm/min overnight. Then, the XAD-16 resins absorbing secondary metabolites was washed with distilled water and eluted with MeOH-H_2_O in gradient from 20% to 100% to give four major fractions (I–IV). Fr. I, eluted with 20% MeOH-H_2_O, was subject to gradient reverse-phase ODS-C_18_ CC eluting with a step gradient (10–50% methanol in water) to obtain 15 sub-fractions (Ia to Io). Sub-frs. Ie and Ig were further separated on semi-preparative reverse-phase HPLC (3 mL/min, detector UV *λ*_max_ 210 nm, CH_3_CN-H_2_O, 5–20% in gradient, 0–50 min) to give **6** (5.9 mg, RT 21.2 min) from Ie and **5** (2.0 mg, RT 24.4 min) and **3** (2.7 mg, RT 25.7 min) from Ig, respectively. Similarly, **2** (1.4 mg, RT 40.1 min), **1** (15.6 mg, RT 36.7 min), and **4** (11.6 mg, RT 39.2 min) were further purified from sub-frs. H, I, and K by semi-preparative reverse-phase HPLC (3 mL/min, detector UV *λ*_max_ 210 nm, CH_3_CN-H_2_O 8:92, MeOH-H_2_O 18:82, and MeOH-H_2_O 20:80, respectively).

D6-*O*-acetyl-acarviostatin II03 (**1**). White amorphous powder; [*α*]${}_{D}^{23}$ + 93.4 (*c* 0.78, H_2_O); UV (H_2_O) end absorption; IR (ATR) *ν*_max_ 3295, 2916, 1724, 16341, 1370, 1147, 997 cm^−1^; ^1^H and ^13^C NMR (D_2_O), see Table 1; HRESIMS *m*/*z* [M + H]^+^ 1477.5564 (calcd for C_58_H_97_N_2_O_41_, 1477.5561).

D6-*O*-*β*-hydroxybutyryl-acarviostatin II03 (**2**). White amorphous powder; [*α*]${}_{D}^{23}$ + 41.1 (*c* 0.44, H_2_O); UV (H_2_O) end absorption; IR (ATR) *ν*_max_ 3276, 2928, 1731, 1653, 1400, 1148, 1002 cm^−1^; ^1^H and ^13^C NMR (D_2_O), see Table 1; HRESIMS *m*/*z* [M + H]^+^ 1521.5829 (calcd for C_60_H_101_N_2_O_42_, 1521.5823).

D6-*O*-acetyl-acarviostatin I03 (**3**). White amorphous powder; [*α*]${}_{D}^{23}$ + 198.2 (*c* 0.27, H_2_O); UV (H_2_O) end absorption; IR (ATR) *ν*_max_ 3286, 2923, 1720, 1635, 1367, 1147, 996 cm^−1^; ^1^H and ^13^C NMR (D_2_O), see Table 2; HRESIMS *m*/*z* [M + H]^+^ 1012.3713 (calcd for C_39_H_66_NO_29_, 1012.3715).

D6-*O*-propionyl-acarviostatin I03 (**4**). White amorphous powder; [*α*]${}_{D}^{23}$ + 54.6 (*c* 1.10, H_2_O); UV (H_2_O) end absorption; IR (ATR) *ν*_max_ 3280, 2924, 1720, 1647, 1378, 1260, 1147, 1015, 996 cm^−1^; ^1^H and ^13^C NMR (D_2_O), see Table 2; HRESIMS *m*/*z* [M + H]^+^ 1026.3872 (calcd for C_40_H_68_NO_29_, 1026.3872).

D6-*O*-*β*-Hydroxybutyryl-acarviostatin I03 (**5**). White amorphous powder; [*α*]${}_{D}^{23}$ + 135.4 (*c* 0.70, H_2_O); UV (H_2_O) end absorption; IR (ATR) *ν*_max_ 3270, 2923, 1721, 1635, 1376, 1148, 998 cm^−1^; ^1^H and ^13^C NMR (D_2_O), see Table 2; HRESIMS *m*/*z* [M + H]^+^ 1056.3976 (calcd for C_41_H_70_NO_30_, 1056.3977).

### 3.4. Conversion of Compounds ***1*** and ***2*** to ***6*** and ***3**–**5*** to ***7***

One milligram of material containing predominantly one of the following: **1**–**5** was dissolved in 2 mL of 0.1 M ammonium hydroxide in MeOH-H_2_O (70:30, *v*/*v*). After ca. 30 h at ambient temperature, the sample was analyzed *via* HPLC-ELSD (Figures S102 and S103). The analysis indicated that **1** and **2** had been converted into co-occurring **6**, while **3**–**5** had been changed into module **7**. The structure of their deacyl-products was identified by comparing of their ^1^H-NMR spectra (Figures S104 and S106) with those reported in the literature [11]. The elution system used for analytical HPLC-ELSD was as follows: 0–30 min, 3–50% methanol in water, 1 mL/min. The retention times for **1**–**7** were 17.1, 16.7, 15.9, 19.2, 15.5, 12.6, and 10.0 min, respectively.

### 3.5. Biological Activity

Porcine pancreatic *α*-amylase inhibitory (PPA) inhibitory activity assay was carried out reference to the procedure previously described [15]. In brief, 5 μL of enzyme solution (PPA, 50 U/mL) and 10-fold series diluted test sample solution (inhibitors, dissolved in distilled water) were mixed and pre-incubated. 100 μL of 0.2% soluble starch (substrate, dissolved in 100 mM phosphate buffer, pH 6.9) was added and incubated at 20 °C for 30 min. The reaction was terminated by the addition of 20 μL 1 M HCl. The remaining starch was quantified by using the following method: 100 μL of the reaction solution was mixed with 25 μL of Lugol’s iodine solution. The absorption was measured at 630 nm in the 96-well plates. Acarbose, an antidiabetic drug, was used as a positive control and averages of three replicates were calculated. The data were imported into GraphPad Prism version 5.0 (GraphPad Software, San Diego, CA, USA, www.graphpad.com) and the IC_50_ values were calculated by using a standard dose response curve fitting.

## 4. Conclusions

In summary, five new rare acylated aminooligosaccharides, D6-*O*-acetyl-acarviostatin II03 (**1**), D6-*O*-*β*-hydroxybutyryl-acarviostatin II03 (**2**), D6-*O*-acetyl-acarviostatin I03 (**3**), D6-*O*-propionyl-acarviostatin I03 (**4**), and D6-*O*-*β*-hydroxybutyryl-acarviostatin I03 (**5**), along with one known related analogue, acarviostatin II03 (**6**), were obtained from marine *Streptomyces* sp. HO1518. All new compounds have shown more potent inhibitory activities against PPA than the clinical drug acarbose, indicating that the marine microorganisms belonging to genus *Streptomyces* are still a rich source for discovering diverse new bioactive natural products that could be used as drug lead toward II-type diabetes.

## Figures and Tables

**Table 1:** ^1^H and ^13^C NMR Data ^a^ for **1**, **2** and **6** in D_2_O ^b^.

No.	1 ^c^		2 ^c^		6	
	*δ*_C_, Type	*δ*_H_ (*J* in Hz)	*δ*_C_, Type	*δ*_H_ (*J* in Hz)	*δ*_C_, Type	*δ*_H_ (*J* in Hz)
**A**1*α*	94.8, CH	5.23, d (3.5)	94.8, CH	5.23, d (3.5)	94.8, CH	5.23, d (3.5)
**A**2*α*	74.2, CH	3.57, m	74.2, CH	3.57, m	74.1, CH	3.57, m
**A**3*α*	76.1, CH	3.98, m	76.0, CH	3.98, m	76.0, CH	3.98, m
**A**4*α*	79.6, CH	3.66, m	79.6, CH	3.66, m	79.6, CH	3.66, m
**A**5*α*	72.8, CH	3.94, m	72.8, CH	3.94, m	72.8, CH	3.94, m
**A**6*α*	63.3, CH_2_	3.84, m	63.3, CH_2_	3.84, m	63.3, CH_2_	3.84, m
**A**1*β*	98.6, CH	4.66, d (8.0)	98.7, CH	4.66, d (8.0)	98.6, CH	4.66, d (8.0)
**A**2*β*	76.9, CH	3.28, dd (9.5, 8.0)	76.7, CH	3.28, dd (9.5, 8.0)	76.8, CH	3.28, dd (9.5, 8.0)
**A**3*β*	79.1, CH	3.78, m	79.1, CH	3.77, m	79.0, CH	3.77, m
**A**4*β*	79.7, CH	3.66, m	79.7, CH	3.66, m	79.7, CH	3.66, m
**A**5*β*	77.4, CH	3.60, m	77.4, CH	3.60, m	77.4, CH	3.60, m
**A**6*β*	63.3, CH_2_	3.91, m	63.4, CH_2_	3.91, m	63.3, CH_2_	3.91, m
**B**1	102.5, CH	5.41, d (3.5)	102.5, CH	5.41, d (3.5)	102.5, CH	5.41, d (3.5)
**B**2	74.4, CH	3.63, m	74.4, CH	3.63, m	74.4, CH	3.63, m
**B**3	76.2, CH	3.96, m	76.2, CH	3.96, m	76.2, CH	3.96, m
**B**4	79.9, CH	3.67, m	79.9, CH	3.67, m	79.9, CH	3.67, m
**B**5	74.0, CH	3.85, m	74.0, CH	3.85, m	74.0, CH	3.85, m
**B**6	63.4, CH_2_	3.84, m	63.4, CH_2_	3.84, m	63.4, CH_2_	3.84, m
**C**1	102.5, CH	5.41, d (3.5)	102.5, CH	5.41, d (3.5)	102.5, CH	5.41, d (3.5)
**C**2	74.4, CH	3.63, m	74.4, CH	3.63, m	74.4, CH	3.63, m
**C**3	76.2, CH	3.96, m	76.2, CH	3.96, m	76.2, CH	3.96, m
**C**4	79.9, CH	3.67, m	79.9, CH	3.67, m	79.9, CH	3.67, m
**C**5	74.0, CH	3.85, m	74.0, CH	3.85, m	74.0, CH	3.85, m
**C**6	63.4, CH_2_	3.84, m	63.5, CH_2_	3.84, m	63.4, CH_2_	3.84, m
**D**1	102.5, CH	5.41, d (3.5)	102.5, CH	5.40, d (3.5)	102.5, CH	5.41, d (3.5)
**D**2	74.4, CH	3.66, m	74.4, CH	3.66, m	74.4, CH	3.66, m
**D**3	76.2, CH	3.95, m	76.2, CH	3.96, m	76.2, CH	3.96, m
**D**4	80.7, CH	3.66, m	80.8, CH	3.66, m	79.8, CH	3.66, m
**D**5	71.7, CH	4.06, m	71.8, CH	4.07, m	74.0, CH	3.85, m
**D**6a	66.5, CH_2_	4.43, dd (12.5, 2.0)	66.5, CH_2_	4.50, dd (12.5, 2.0)	63.5, CH_2_	3.84, m
**D**6b		4.24, m		4.26, m		
**E**1	103.4, CH	5.30, d (3.5)	103.4, CH	5.29, d (3.5)	102.7, CH	5.33, d (3.5)
**E**2	74.1, CH	3.59, m	74.1, CH	3.60, m	74.1, CH	3.60, m
**E**3	75.8, CH	3.63, m	75.8, CH	3.63, m	75.8, CH	3.63, m
**E**4	67.1, CH	2.46, t (9.0)	67.1, CH	2.47, t (9.0)	67.0, CH	2.47, t (9.0)
**E**5	72.6, CH	3.74, m	72.6, CH	3.76, m	72.5, CH	3.74, m
**E**6	20.2, CH_3_	1.31, d (6.0)	20.2, CH_3_	1.32, d (6.0)	20.2, CH_3_	1.32, d (6.0)
**F**1	57.8, CH	3.53, m	57.9, CH	3.53, m	57.9, CH	3.53, m
**F**2	72.5, CH	3.80, m	72.5, CH	3.80, m	72.4, CH	3.80, m
**F**3	73.6, CH	4.16, m	73.7, CH	4.16, m	73.5, CH	4.16, m
**F**4	79.1, CH	4.24, d (6.4)	79.1, CH	4.23, d (6.4)	78.9, CH	4.23, d (6.4)
**F**5	139.4, C		139.3, C		139.3, C	
**F**6a	64.9, CH_2_	4.22, m	64.9, CH_2_	4.22, m	64.9, CH_2_	4.22, m
**F**6b		4.14, m		4.14, m		4.14, m
**F**7	129.2, CH	5.98, d (4.5)	129.2, CH	5.98, d (4.5)	129.1, CH	5.98, d (4.5)
**G**1	100.4, CH	5.38, d (4.0)	100.4, CH	5.38, d (4.0)	100.4, CH	5.38, d (4.0)
**G**2	74.3, CH	3.63, m	74.3, CH	3.63, m	74.4, CH	3.63, m
**G**3	76.4, CH	3.92, m	76.4, CH	3.92, m	76.3, CH	3.92, m
**G**4	79.9, CH	3.61, m	79.9, CH	3.61, m	79.9, CH	3.61, m
**G**5	74.0, CH	3.90, m	74.0, CH	3.90, m	74.0, CH	3.90, m
**G**6	63.5, CH_2_	3.86, m	63.6, CH_2_	3.86, m	63.5, CH_2_	3.86, m
**H**1	102.8, CH	5.33, d (3.5)	102.8, CH	5.34, d (3.5)	102.7, CH	5.33, d (3.5)
**H**2	74.2, CH	3.58, m	74.2, CH	3.59, m	74.1, CH	3.58, m
**H**3	75.4, CH	3.61, m	75.4, CH	3.63, m	75.4, CH	3.61, m
**H**4	67.8, CH	2.48, t (9.0)	67.8, CH	2.49, t (9.0)	67.8, CH	2.48, t (9.0)
**H**5	72.5, CH	3.76, m	72.5, CH	3.78, m	72.3, CH	3.76, m
**H**6	20.2, CH_3_	1.35, d (6.0)	20.2, CH_3_	1.36, d (6.0)	20.2, CH_3_	1.35, d (6.0)
**I**1	58.8, CH	3.54, m	58.9, CH	3.54, m	58.8, CH	3.54, m
**I**2	75.5, CH	3.67, m	75.5, CH	3.67, m	75.5, CH	3.67, m
**I**3	75.6, CH	3.77, m	75.6, CH	3.77, m	75.6, CH	3.77, m
**I**4	73.8, CH	4.05, d (6.4)	73.8, CH	4.05, d (6.4)	73.7, CH	4.05, d (6.4)
**I**5	141.8, C		141.8, C		141.8, C	
**I**6a	64.5, CH_2_	4.23, m	64.5, CH_2_	4.24, m	64.4, CH_2_	4.23, m
**I**6b		4.12, m		4.12, m		4.12, m
**I**7	126.6, CH	5.90, dd (5.0, 1.0)	126.7, CH	5.91, dd (5.0, 1.0)	126.6, CH	5.91, dd (5.0, 1.0)
**D**6-*O*-acetyl	177.0, C		-	-	-	-
	23.1, CH_3_	2.17 (3H, s)	-	-	-	-
**D**6-*O*-2-hydroxybutyryl	-	-	176.6, C		-	-
	-	-	45.9, CH_2_	2.67, dd (15.0, 4.5) 2.59, dd (15.0, 8.0)	-	-
	-	-	67.3, CH	4.27, m	-	-
	-	-	24.8, CH_3_	1.26, d (6.5)	-	-

^a 1^H NMR data recorded at 500 MHz and ^13^C NMR data recorded at 125 MHz, 298 K, 18.0 mg/mL for **1** and 2.8 mg/mL for **2**; ^b^ Unstable in D_2_O; ^c^ Every spin system in rings **A**–**I** was acquired by 1D-selective TOCSY experiments with the same NMR parameters. Meanwhile, although the coupling constants and concentration were identical in rings **E** and **H**, the intensity of the spin systems in both rings were different; one (ring **H**) was strong while the other (ring **E**) was relatively weak. This phenomenon maybe greatly related to their locations in the molecules.

**Table 2:** ^1^H and ^13^C NMR Data for **3**–**5** in D_2_O.

No.	3 ^a^^,c^		4 ^a^^,c^		5 ^b^^,c^		7 [11]
	*δ*_C_, Type	*δ*_H_ (*J* in Hz)	*δ*_C_, Type	*δ*_H_ (*J* in Hz)	*δ*_C_, Type	*δ*_H_ (*J* in Hz)	*δ*_C_, Type
**A**1*α*	94.7, CH	5.23, d (3.5)	94.8, CH	5.23, d (3.5)	94.8, CH	5.24, d (3.5)	93.0
**A**2*α*	74.2, CH	3.58, m	74.1, CH	3.58, m	74.2, CH	3.58, m	72.6
**A**3*α*	76.0, CH	3.98, m	76.0, CH	3.98, m	76.0, CH	3.98, m	74.4
**A**4*α*	79.5, CH	3.66, m	79.6, CH	3.66, m	79.5, CH	3.67, m	79.3
**A**5*α*	72.8, CH	3.94, m	72.8, CH	3.94, m	72.8, CH	3.95, m	71.0
**A**6*α*	63.3, CH_2_	3.84, m	63.2, CH_2_	3.84, m	63.2, CH_2_	3.84, m	61.5
**A**1*β*	98.6, CH	4.66, d (8.0)	98.6, CH	4.66, d (8.0)	98.6, CH	4.66, d (8.0)	96.8
**A**2*β*	76.9, CH	3.28, dd (9.5, 8.0)	76.9, CH	3.28, dd (9.5, 8.0)	76.9, CH	3.28, dd (9.5, 8.0)	75.1
**A**3*β*	79.1, CH	3.77, m	79.0, CH	3.78, m	79.1, CH	3.78, m	77.3
**A**4*β*	79.6, CH	3.66, m	79.7, CH	3.66, m	79.6, CH	3.66, m	77.8
**A**5*β*	77.4, CH	3.60, m	77.4, CH	3.60, m	77.4, CH	3.60, m	75.6
**A**6*β*	63.5, CH_2_	3.91, m	63.5, CH_2_	3.91, m	63.5, CH_2_	3.91, m	61.5
**B**1	102.5, CH	5.41, d (3.5)	102.5, CH	5.41, d (3.5)	102.5, CH	5.41, d (3.5)	100.7
**B**2	74.4, CH	3.63, m	74.4, CH	3.63, m	74.4, CH	3.63, m	72.6
**B**3	76.2, CH	3.96, m	76.2, CH	3.96, m	76.2, CH	3.96, m	74.4
**B**4	79.8, CH	3.67, m	79.9, CH	3.67, m	79.8, CH	3.67, m	78.0
**B**5	74.0, CH	3.85, m	74.0, CH	3.85, m	74.0, CH	3.85, m	72.2
**B**6	63.4, CH_2_	3.84, m	63.2, CH_2_	3.84, m	63.4, CH_2_	3.84, m	61.5
**C**1	102.5, CH	5.41, d (3.5)	102.5, CH	5.41, d (3.5)	102.5, CH	5.41, d (3.5)	100.7
**C**2	74.4, CH	3.63, m	74.4, CH	3.63, m	74.4, CH	3.63, m	72.6
**C**3	76.2, CH	3.96, m	76.2, CH	3.96, m	76.2, CH	3.96, m	74.4
**C**4	79.8, CH	3.67, m	79.9, CH	3.67, m	79.8, CH	3.67, m	78.0
**C**5	74.0, CH	3.85, m	74.0, CH	3.85, m	74.0, CH	3.85, m	72.2
**C**6	63.5, CH_2_	3.84, m	63.2, CH_2_	3.84, m	63.4, CH_2_	3.84, m	61.5
**D**1	102.5, CH	5.41, d (3.5)	102.5, CH	5.41, d (3.5)	102.5, CH	5.41, d (3.5)	100.7
**D**2	74.4, CH	3.67, m	74.4, CH	3.67, m	74.4, CH	3.66, m	72.6
**D**3	76.2, CH	3.95, m	76.2, CH	3.96, m	76.2, CH	3.96, m	74.4
**D**4	80.7, CH	3.67, m	80.7, CH	3.67, m	80.8, CH	3.66, m	78.0
**D**5	71.7, CH	4.05, m	71.8, CH	4.05, m	71.8, CH	4.06, m	72.2
**D**6a	66.5, CH_2_	4.43, dd (12.5, 2.0)	66.3, CH_2_	4.44, dd (12.5, 2.0)	66.5, CH_2_	4.50, dd (12.5, 2.0)	61.5
**D**6b		4.24, brd (12.5)		4.24, brd (12.5)		4.26, brd (12.5)	
**E**1	103.4, CH	5.30, d (3.5)	103.4, CH	5.29, d (3.5)	103.4, CH	5.28, d (3.5)	101.0
**E**2	74.4, CH	3.59, m	74.2, CH	3.58, m	74.2, CH	3.59, m	70.7
**E**3	75.47, CH	3.62, m	75.6, CH	3.62, m	75.6, CH	3.62, m	73.8
**E**4	67.7, CH	2.48, t (9.0)	67.9, CH	2.46, t (9.0)	67.9, CH	2.47, t (9.0)	66.0
**E**5	72.4, CH	3.72, m	72.7, CH	3.71, m	72.6, CH	3.73, m	70.7
**E**6	20.2, CH_3_	1.32, d (6.0)	20.2, CH_3_	1.31, d (6.0)	20.2, CH_3_	1.32, d (6.0)	18.4
**F**1	58.8, CH	3.54, m	58.8, CH	3.53, m	58.9, CH	3.54, m	57.1
**F**2	75.52, CH	3.66, m	75.7, CH	3.66, m	75.7, CH	3.66, m	74.0
**F**3	75.8, CH	3.76, m	75.8, CH	3.77, m	75.8, CH	3.76, m	73.8
**F**4	73.5, CH	4.05, d (6.4)	73.7, CH	4.05, d (6.4)	73.9, CH	4.04, d (6.4)	72.2
**F**5	142.3, C		141.9, C		141.9, C		140.1
**F**6a	64.4, CH_2_	4.23, brd (13.6)	64.4, CH_2_	4.24, brd (13.6)	64.4, CH_2_	4.23, brd (13.6)	62.6
**F**6b		4.12, brd (13.6)		4.12, brd (13.6)		4.12, brd (13.6)	
**F**7	126.1, CH	5.90, dd (5.0, 1.0)	126.6, CH	5.91, dd (5.0, 1.0)	126.5, CH	5.90, dd (5.0, 1.0)	124.8
**D**6-*O*-acetyl	177.0, C		-	-	-	-	-
	23.1, CH_3_	2.16, s	-	-	-	-	-
**D**6-*O*-propionyl	-	-	180.3, C		-	-	-
	-	-	30.0, CH_2_	2.48, q (7.5)	-	-	-
	-	-	11.1, CH_3_	1.13, t (7.5)	-	-	-
**D**6-*O*-2-hydroxy-butyryl	-	-	-	-	176.6, C		-
	-	-	-	-	45.9, CH_2_	2.67, dd (15.0, 4.5) 2.59, dd (15.0, 8.0)	-
	-	-	-	-	67.3, CH_2_	4.28, m	-
	-	-	-	-	24.8, CH_3_	1.26, d (6.0)	-

^a^ Measured at 500 MHz for ^1^H and 125 MHz for ^13^C NMR, 298 K, 6.0 mg/mL for **3** and 20.0 mg/mL for **4**; ^b^ Measured at 400 MHz for ^1^H and 100 MHz for ^13^C NMR, 298 K, 4.0 mg/mL for **5**; ^c^ unstable in D_2_O.

**Table 3:** Pancreatic *α*-amylase (PPA) inhibitory activity data for **1**–**6**.

Test Samples	IC_50_ (μM)	Test samples	IC_50_ (μM)
**1**	0.029 ± 0.001	**3**	0.693 ± 0.097
**2**	0.049 ± 0.016	**4**	0.625 ± 0.024
**6**	0.029 ± 0.004	**5**	0.495 ± 0.063
acarbose ^a^	15.7		

^a^ Positive control.

## Supplementary Materials

The following are available online at https://zenodo.org/record/1471953#.W9LTdvabyUm. HRESIMS, HRESIMS/MS, ^1^H, ^13^C NMR, DEPT, ^1^H-^1^H COSY, 1D-selective TOCSY, 2D TOCSY, HSQC, HSQC-TOCSY, HMBC, ROESY, IR, and UV spectra and [*α*]_D_ data of the new acylated aminooligosaccharides **1**–**5**; ^1^H NMR and HPLC-ELSD of the alkaline hydrolysis products of **1** and **2** and **3**–**5**.

## References

[1] Cantarel B.L., Coutinho P.M., Rancurel C., Bernard T., Lombard V., Henrissat B. (2009). The carbohydrate-active enzymes database (cazy): An expert resource for glycogenomics. Nucleic. Acids Res..

[2] Yoon S.H., Robyt J.F. (2003). Study of the inhibition of four *α*-amylases by acarbose and its 4^IV^-*α*-maltohexaosyl and 4^IV^-*α*-maltododecaosyl analogues. Carbohydr. Res..

[3] De Sales P.M., de Souza P.M., Dartora M., Resck I.S., Simeoni L.A., Fonseca-Bazzo Y.M., de Oliveira Magalhães P., Silveira D. (2017). *Pouteria torta* epicarp as a useful source of *α*-amylase inhibitor in the control of type 2 diabetes. Food Chem. Toxicol..

[4] Schmidt D.D., Frommer W., Junge B., Müller L., Wingender W., Truscheit E., Schäfer D. (1977). *α*-Glucosidase inhibitors. New complex oligosaccharides of microbial origin. Naturwissenschaften.

[5] Truscheit E., Frommer W., Junge B., Müller L., Schmidt D.D., Wingender W. (1981). Chemistry and biochemistry of *α*-glucosidase inhibitors. Angew. Chem. Int. Ed. Engl..

[6] Yokose K., Ogawa K., Sano T., Watanabe K., Maruyama H.B., Suhara Y. (1983). New *α*-amylase inhibitor, trestatins I. Isolation characterization and biological activities of trestatins A, B, and C. J. Antibiot..

[7] Yokose K., Ogawa K., Suzuki Y., Umeda I., Suhara Y. (1983). New *α*-amylase inhibitor, trestatins II. Structure determination of trestatins A, B and C. J. Antibiot..

[8] Shi D.-Y., Zhong D.-F., Chen X.-Y. (2001). Profiling of isovalertatin family aminooligosaccharides extracted from the culture of *Streptomyces luteogriseus* by using liquid chromatography/electrospray ionization mass spectrometry. Anal. Chem..

[9] Zhong D., Si D.-Y., He W.-Y., Zhao L.-M., Xu Q.-M. (2001). Structural revision of isovalertatins M03, M13, and M23 isolated from the culture of *Streptomyces luteogriseus*. Carbohydr. Res..

[10] Si D.-Y., Zhong D.-F., Xu Q.-M. (2001). Two butylated aminooligosaccharides isolated from the culture filtrate of *Streptomyces luteogriseus*. Carbohydr. Res..

[11] Geng P., Qiu F., Zhu Y.-Y., Bai G. (2008). Four acarviosin-containing oligosaccharides identified from *Streptomyces coelicoflavus* ZG0656 are potent inhibitors of *α*-amylase. Carbohydr. Res..

[12] Geng P., Bai G. (2008). Two novel aminooligosaccharides isolated from the culture of *Streptomyces coelicoflavus* ZG0656 as potent inhibitors of *α*-amylase. Carbohydr. Res..

[13] Geng P., Meng X.-S., Bai G., Luo G.-A. (2008). Profiling of acarviostatin family secondary metabolites secreted by *Streptomyces coelicoflavus* ZG0656 using ultraperformance liquid chromatography coupled with electrospray ionization mass spectrometry. Anal. Chem..

[14] Geng P., Sun T., Zhong Q.-P., Li X.-X., Shi L.-Y., Bai F., Bai G. (2013). Two novel potent *α*-amylase inhibitors from the family of acarviostatins isolated from the culture of *Streptomyces coelicoflavus* ZG0656. Chem. Biodivers..

[15] Wang L.-Q., Cui Q.-X., Hou Y.-Y., Bai F., Sun J.-X., Cao X.-F., Liu P., Jiang M., Bai G. (2013). An integrated strategy of ultra-high-performance liquid chromatography/quadrupole-time-of-flight mass spectrometry and virtual screening for the identification of *α*-glucosidase inhibitors in acarviostatin-containing complex. J. Chromatogr. A.

[16] Meng P., Guo Y.-Q., Zhang Q., Hou J., Bai F., Geng P., Bai G. (2011). A novel amino-oligosaccharide isolated from the culture of *Streptomyces* strain PW638 is a potent inhibitor of *α*-amylase. Carbohydr Res..

[17] Qin X.-H., Ren L.-M., Yang X., Bai F., Wang L.-L., Geng P., Bai G., Shen Y.-Q. (2011). Structures of human pancreatic *α*-amylase in complex with acarviostatins: Implications for drug design against type II diabetes. J. Struct. Biol..

[18] Geng P., Bai G., Shi Q., Zhang L., Gao Z.-H., Zhang Q. (2009). Taxonomy of the *Streptomyces* strain ZG0656 that produces acarviostatin *α*-amylase inhibitors and analysis of their effects on blood glucose levels in mammalian systems. J. Appl. Microbiol..

[19] Xiong Z.-Q., Liu Q.-X., Pan Z.-L., Zhao N., Feng Z.-X., Wang Y. (2015). Diversity and bioprospecting of culturable actinomycetes from marine sediment of the Yellow Sea, China. Arch. Microbiol..

[20] Xiong Z.-Q., Wang J.-F., Hao Y.-Y., Wang Y. (2013). Recent advances in marine microbial natural products discovery and development. Mar. Drugs.

[21] Xiong Z.-Q., Wang Y. (2012). Draft genome sequence of the marine *Streptomyces* sp. strain AA1529, isolated from the Yellow Sea. J. Bacteriol..

[22] Xiong Z.-Q., Wang Y. (2012). Draft genome sequence of marine-derived *Streptomyces* sp. strain AA0539, isolated from the Yellow Sea, China. J. Bacteriol..

[23] Ono M., Oda S., Yasuda S., Mineno T., Okawa M., Kinjo J., Miyashita H., Yoshimitsu H., Nohara T., Miyahara K. (2017). Acylated glycosidic acid methyl ester generated from the convolvulin fraction of Rhizoma Jalapae Braziliensis by treatment with indium (III) chloride in methanol. Chem. Pharm. Bull..

[24] Yu L., Trujillo M.E., Miyanaga S., Saiki I., Igarashi Y. (2014). Campechic acids A and B: anti-invasive polyether polyketides from a soil-derived *Streptomyces*. J. Nat. Prod..

